# “…because the social work never ends”: a qualitative study exploring how NGOs responded to emerging needs while upholding responsibility to HIV prevention and treatment during the war in Ukraine

**DOI:** 10.1002/jia2.26309

**Published:** 2024-07-19

**Authors:** Lisa Lazarus, Leigh M McClarty, Nicole Herpai, Daria Pavlova, Tatiana Tarasova, Anna Gnatenko, Tetiana Bondar, Robert Lorway, Marissa L Becker, the Dynamics Study Team

**Affiliations:** ^1^ Institute for Global Public Health Rady Faculty of Health Sciences University of Manitoba Winnipeg Canada; ^2^ Department of Community Health Sciences Max Rady College of Medicine Rady Faculty of Health Sciences University of Manitoba Winnipeg Canada; ^3^ Ukrainian Institute for Social Research after Oleksandr Yaremenko Kyiv Ukraine

**Keywords:** HIV, NGO, key populations, conflict, war, Ukraine

## Abstract

**Introduction:**

Since the onset of the Russian invasion on 24 February 2022, the health system in Ukraine has been placed under tremendous pressure, with damage to critical infrastructure, large losses of human resources, restricted mobility and significant supply chain interruptions. Based on a longstanding partnership between the Ukrainian Institute for Social Research after Oleksandr Yaremenko (UISR after O. Yaremenko) and the Institute for Global Public Health at the University of Manitoba, we explore the impact of the full‐scale war on non‐governmental organizations (NGOs, including charitable organizations) providing services for key population groups in Ukraine.

**Methods:**

We conducted in‐depth qualitative interviews with key representatives from NGOs working with key population groups (i.e., people living with HIV, sex workers, men who have sex with men, people who inject drugs and transgender people) throughout Ukraine. Members of the UISR after O. Yaremenko research team recruited participants from organizations working at national, regional and local levels. The research team members conducted 26 interviews (22 with women and four with men) between 15 May and 7 June 2023. Interviews were conducted virtually in Ukrainian and interpretively analysed to draw out key themes.

**Results:**

Applying Roels et al.’s notion of “first responders”, our findings explore how the full‐scale war personally and organizationally impacted workers at Ukrainian NGOs. Despite the impacts to participants’ physical and mental health, frontline workers continued to support HIV prevention and treatment while also responding to the need for humanitarian aid among their clients and the wider community. Furthermore, despite inadequate pay and compensation for their work, frontline workers assumed additional responsibilities, thereby exceeding their normal workload during the extraordinary conditions of war.

**Conclusions:**

NGOs play a vital role as responders, adapting their services to meet the emergent needs of communities during structural shocks, such as war. There is an urgent need to support NGOs with adequate resources for key population service delivery and to increase support for their important role in humanitarian aid.

## 1 INTRODUCTION

Russia's invasion of Ukraine on 24 February 2022, has led to ongoing disruptions to health and social services across the country [1, 2, 3]. In Ukraine, HIV treatment is funded by the state, and its provision is centralized through AIDS Centers within each region [4]. At the same time, HIV prevention services are almost exclusively funded through international donors, such as the Global Fund and the United States President's Emergency Plan for AIDS Relief (PEPFAR), and delivered through non‐governmental organizations (NGOs) [5, 6, 7, 8]. NGOs provide HIV prevention services and resources to key populations (sex workers, men who have sex with men, people who inject drugs and transgender people), including harm reduction supplies, condoms and counselling and testing for HIV and other sexually transmitted and blood‐borne infections [6, 9]. During the Donbas war (2014–2022), Ukraine began a multi‐year, multi‐pronged healthcare reform process [10, 11], transitioning funding for HIV care and treatment from international donors to the government in 2021 [4, 12]. However, with the war came another influx of international donor support, with some funding earmarked for HIV treatment and prevention [12]. In 2022, the Global Fund and PEPFAR provided 85% of the HIV budget, and in 2023, the Ukrainian government provided no funding for HIV. In the first year of the war, foreign donors provided in excess of 38 million USD in emergency funding for HIV prevention and treatment services [13, 14, 15].

The Ukrainian healthcare system has been under immense pressure since the war, with damage to critical infrastructure, large losses of human resources, restricted mobility and significant supply chain interruptions [12, 16, 17, 18, 19]. As of 5 January 2024, the World Health Organization registered 1435 attacks on healthcare facilities, including 218 injuries and 112 deaths of both patients and personnel [3]. Approximately 38 out of 392 antiretroviral therapy (ART) sites have shut down, and laboratories in the occupied Donetsk and Luhansk oblasts have discontinued their operations [17, 20]. The displacement of medical staff and social workers has strained the human resources available for providing HIV treatment and prevention [16, 17].

At the end of 2021, an estimated 245,000 people were living with HIV (people living with HIV [PLHIV]) in Ukraine, 152,000 of whom were on ART [21]. As of December 2023, the registered number of PLHIV in Ukraine was 158,803, and according to the Public Health Center of the Ministry of Health of Ukraine data, as of November 2023, there were 121,820 people receiving ART [22]. While current HIV incidence and prevalence data are not available, reports from 2021 indicated an HIV prevalence of 0.94% among adults 15 to 49 [21]. In the same year, the HIV prevalence was 3.1% among sex workers and 3.9% among men who have sex with men. Data from 2020 showed an HIV prevalence of 20.3% among people who use injection drugs and 1.7% among transgender people [21]. The supply of enough ART during the war has been assured by partners including the Global Fund, the Joint United Nations Programme on HIV/AIDS (UNAIDS), the United States Agency for International Development (USAID) and PEPFAR [13, 23, 24]. Despite adequate supplies, approximately 30% of PLHIV reported disruptions to treatment since the onset of the war [17]. Historically, there have been higher rates of HIV in eastern Ukraine [25], which currently includes Russian‐occupied zones [26] and has resulted in missing data on whether PLHIV are receiving the care that they require [20]. Even amidst the full‐scale war, access to ART was ensured for all who required treatment within government‐controlled territories [27].

NGOs play an important role in providing HIV prevention, treatment and care in Ukraine, especially for key populations [9, 28]. NGOs that focus on providing services to people who use injection drugs have significantly expanded access to HIV prevention, care and treatment, connecting key populations to essential HIV‐related services [29, 30, 31]. Notably, in 2022, NGOs managed to achieve substantial coverage of prevention services. Despite the war, 62% of registered NGO clients received prevention services, and approximately 84% were tested for HIV [32]. In 2023, collaboration between 88 NGOs providing HIV services and the Public Health Center worked to further strengthen care and support efforts for PLHIV and prevention services for key populations. These NGOs are strategically distributed across various regions, with concentrations in Kyiv, Dnipro and Odesa oblasts. Among the 88 NGOs, 58 focus on HIV prevention programmes for people who inject drugs, 33 for sex workers and 33 for men who have sex with men. Additionally, 10 organizations provide prevention services for transgender people, and six provide services to incarcerated populations, with multiple organizations often serving more than one key population [32].

Drawing on a longstanding partnership between the Ukrainian Institute for Social Research after Oleksandr Yaremenko (UISR after O. Yaremenko) and the Institute for Global Public Health at the University of Manitoba, we aimed to explore the impact of the war on NGOs providing HIV prevention and treatment services for key population groups across the country.

## 2 METHODS

This study builds on the work of the *Dynamics Study*, a mixed‐methods project exploring the impact of conflict on sex work and the HIV/hepatitis C (HCV) epidemics in Dnipro, Ukraine [33]. After the COVID‐19 pandemic and the onset of full‐scale war, our team refocused study activities towards conducting a series of in‐depth, key informant interviews with representatives from NGOs working with key population groups across Ukraine. Interviews explored how participants’ programmes were impacted and adapted during the war. The UISR after O. Yaremenko research team enrolled participants from NGOs working at national, regional and local levels. Participants were aged 18 years and older, had at least 2 years’ experience providing services to key population groups and had experience providing services during the war in Ukraine (i.e., since February 2022). More broadly, organizations were selected to ensure representation of (i) regions across the country, (ii) service provisions for different key population groups, (iii) level of operation (national, regional and local) and (iv) types of services (social‐medical and social).

A trained team of interviewers conducted 26 interviews with individuals representing 24 organizations between 15 May and 7 June 2023. Twenty‐two of the participants were women and four were men and included: five interviews with organizations that work nationally; three interviews with organizations that work in central Ukraine; three interviews with organizations that work in the West; eight interviews with organizations in the East, including cities that were under Russian occupation and have experienced both wars (2014 and 2022); two interviews with organizations that work in the North; and five interviews with organizations in the South, including cities that were under Russian occupation after February 2022. Twenty‐four of the participants represented NGOs (including charitable organizations), and two were from governmental organizations. In discussion with local partners, we only identify participants by the region in which they work to protect their confidentiality and anonymity during an ongoing war across the country. Questions explored how participants were impacted by the war; how their organizations were impacted and how their work might have changed; how service provision was affected, especially as it related to services for key populations; and how these changes have impacted their clients. Interviews were conducted virtually in Ukrainian, recorded and transcribed, and then translated into English.

All participants provided verbal, informed consent to participate in the study, and ethical approvals were obtained from the Health Research Ethics Board at the University of Manitoba, Canada, and the Ethical Review Committee of the Sociological Association of Ukraine.

### 2.1 Analysis

We borrow the concept of “first responders” from Roels et al.’s study on the responses of community‐based organizations working with youth in Amsterdam and New York City during the COVID‐19 pandemic [34]. As Roels and colleagues note: “…mechanisms of well‐being are foundationally relational. They depend upon ‘connectivity’, the dense connections that develop between partner organizations and the communities and participants they serve” [p.2].

We draw on the notion of first responders to evoke how Ukrainian NGOs are often first to respond to what Friedman et al. have called “Big Events” [35, 36, 37]—moments of war, pandemics and political change—due to their “connectivity” to communities and their “nimbleness” to quickly respond and adapt their services as needed [34]. The concept of first responders informed our thematic analysis exploring the impact of the war on NGOs serving key populations across Ukraine.

The first two authors (LL and LMM) independently reviewed and inductively coded the first six transcripts to develop a simplified coding scheme aimed at sharing findings back with participant organizations and government partners to help inform their work. The first author (LL) then thematically coded the remaining transcripts using NViVo 12 software, drawing on an interpretivist lens [38]. Preliminary findings were shared and discussed with the research team before finalizing analyses.

## 3 RESULTS

In the sections below, we present our findings on the following themes: (1) how participants and their work were impacted by the escalation of war; (2) how frontline workers continued to support HIV prevention and treatment while also responding to the need for humanitarian aid among the wider community; and (3) how frontline workers went above expected responsibilities, despite inadequate pay and compensation for their work.

### 3.1 Continuing to provide services under the extraordinary conditions of war

Participants spoke of the initial shock of the launch of full‐scale war on 24 February 2022. Despite initial feelings of terror, many participants soon resumed their work responsibilities.

Well, probably like everyone else, I was in shock. There was such a panic at first. Because we really didn't know what was going to happen tomorrow. We didn't know what would happen tomorrow, not only to our organization, to our work, to our clients…. Because those first air sirens were very traumatic…. For the first period, I don't know what period, I don't remember now, we didn't go to work. But a person adapts to everything. You start to get used to everything. You realize that you need to move on. Slowly, slowly, we began to recover and resume our activities and services. Because our clients still have needs…. (Microregion: West)

Disruptions to services appeared to last for incredibly short durations, as staff returned to work to respond to their clients’ needs, and responsibilities were reallocated among staff who stayed. Despite the impact to their own realities, participants spoke of their responsibility to return to work as a primary source of support to their clients, who reached out to them for information, as illustrated in the quotation below:

… but despite the fact that we did not know whether we would be paid for our services, we still went to work, because already a large number of people started calling from the very first day in complete confusion as to how everything will happen next. How will it be possible to take medicine in the future? Where will it be possible to take it? Will AIDS Centers and trust offices work? Will it be possible to take these medicines for free? For what period of time? For what period? How will the treatment of HIV‐positive people take place, or ART drugs will be purchased? … I tried to assure the people who contacted me that everything will be fine, that nothing will be interrupted, that the treatment will continue as it was…. (East)

NGO workers returned to work, at their own risk and amidst grave uncertainties, to adapt services to the current reality of war while facing staffing shortages, shelling, bombings and at times, destroyed workplaces. Some organizations operating in cities that were attacked and occupied moved their services to be based out of non‐occupied cities nearby.

Well, what changed? Life changed very much, for the worse. Because many people left. Those specialists who had worked in the organization for many years and whom we had trained, they left, and the organization became bare. We have to train new specialists now. When we worked during the occupation, it was a daily risk. Now it's a daily risk of being hit by shelling and being killed. It's a daily risk, minute by minute, hour by hour. (South)

NGOs and their staff assumed these personal risks to continue providing their clients with necessary treatments—ranging from ART to opioid substitution therapy (OST) and hormone replacement therapy (HRT)—despite the increased risk of providing services to criminalized populations, including men who have sex with men, people who use drugs and transgender people, under Russian occupation.

### 3.2 Continuing to prioritize HIV prevention and treatment while also responding to humanitarian needs

At the onset of war, NGOs and frontline workers turned their attention towards mobilizing resources to provide humanitarian aid to their clients.

As an organization [name], we did not stop our services for a single day. We worked on February 24…. We added new services immediately to our clients. We communicated very quickly with some donors and donors communicated with us. On February 28, we started paying out financial aid to our clients. To those whom we could, directly to the cards. This is something that [organization name] has never done before, because we did not have such a need. Not even such a need, not even such an opportunity…. (National)

NGOs adapted to meet the basic needs of their clients, tapping in to both their donor and client networks, to provide essential goods such as hygiene products, power banks, clothes, food kits, certificates for groceries and financial aid. Services were expanded beyond key population groups to reach internally displaced people and also included requests for psychological support and replacing lost documents, which are especially necessary for accessing healthcare and medications.

Well, in the first place, it is humanitarian aid, which remains the most common need: food, medicine, especially medicine, hygiene products, means of prevention. In winter, for example, when they started bombing power plants, there was no light, no heat, so we were bombarded with requests: we needed warm blankets, flashlights, power banks. That is, what we all needed here, so did they. And we bought it all, if possible, there: sleeping bags, tents, thermal underwear. Well, we had a very long list of things they needed. Now, of course, it is already warm, and all this has faded into the background. Now more products, medicines, hygiene products and plus, again, housing remain relevant, because most people live in rented apartments…. (National)

While individual priorities shifted towards basic needs and survival during war, NGOs continued to stress the importance of HIV prevention and treatment to their clients. Participants stated that their clients shifted their attention to immediate concerns; however, in their roles as frontline workers, they continued to stress the need for testing and treatment—pointing to the importance of protecting the long‐term health of their clients beyond the immediacy of war.

…. The war has changed the values in each of us, the attitude to life, it is the priority of what life should be given to. And people who had a well‐established life, work, life, they had everything, then they could concentrate some attention on their health. Now it is not a priority, now it is a priority to survive, to eat, and people's heads are full of other things. And people now refuse, ‘nothing hurts me’ and they turn to in extreme cases, when they have acute pains or something. And they don't want to think about HIV, which doesn't bother them at all. Therefore, our specialists and social workers spend more time now motivating people, building commitment to what needs to be done. Because no one has cancelled the epidemics. More time is spent on these explanations. We have more people who refuse our services. This, of course, has a very strong psychological effect on our employees and makes it difficult to work. But it is ok, now it is difficult for everyone in the conditions of war. (East)

Participants spoke of the additional work and stress that went into encouraging clients to continue prioritizing ART to maintain their health. They also described their expanded roles serving as sources of information on accessing treatment for newly displaced people and clients who had relocated, both within and outside of Ukraine. Participants at times kept in touch with these clients and provided information on other organizations they could access for treatment and support. Frontline workers also maintained access to ART through outreach, shifting to mobile clinics, reorienting outreach routes to reflect the realities of curfews and increasing the number of pills distributed at a time.

….A lot of people in the period of time made the decision to leave. And [name] tried to put herself in the position of each person and gave drugs so that a person could go somewhere abroad and not run in search of treatment every day. I tried to give for a month, for two, for three, so that there was a mandatory supply of medicines. I was more or less calm about people. The workload on social workers increased because people were afraid to go to a medical facility during the alarms. An additional, let's say, duty fell on us. There was an obligation not as social workers, but as people who provide help to positive people to send ART therapy.

The same participant goes on to detail how social workers went out of their way to figure out how to mail medications to their clients to ensure they had continued access to treatment:

There was a misunderstanding of how the Nova Poshta [a private courier service] works, there was a misunderstanding of how Ukrposhta [state services] works. There was a certain period of time when the Nova Poshta did not want to take medicines for transportation, for sending, so we had to look for those post offices that agreed to send these medicines. The workers were under a very, very heavy load, I would say…. (East)

NGO workers took on a similar approach to ensure that people who inject drugs had continued access to OST and transgender people continued to have access to HRT. Participants spoke of providing safety information for participants who might be travelling and transporting medicines, at risk to themselves, under Russian invasion to ensure clients’ access to necessary prescriptions was maintained.

I know for sure that they kept in touch with patients who were on the OST sites and with HIV‐positive patients. Communication was maintained because the social work never ends, so you will support me that if the social support of the client has ended, this does not mean that the client has gone somewhere. He will call, communicate, sometimes he does not call the doctor, but calls the social worker, to ask him to go and find out the results of the tests, to go and help him with something else, all this remained…. (Center)

NGOs also maintained access to HIV pre‐exposure prophylaxis (PrEP), HIV testing, naloxone, condoms and harm reduction supplies—demands for which increased among some clients— in order to maintain prevention efforts.

…. In addition, with Alliance [for Public Health], we have a direction on prevention, this is naloxone, which is in great demand. That is, when the war started, it was, well, a cry of the soul, because naloxone was nowhere to be found. The [city] factory was not working, most likely, and there was no way to buy it. Now everything is more settled, we are already… Because the war started—we dug up the remains wherever we could, looking for it. And we have clients, well, it is in great demand. This is their first medical aid while they wait for the ambulance. And every client wants to have an ampoule of naloxone with them. We have a project, that is, they are all informed how to use it, and well… it was in demand…. (Center)

Participants pointed to the response by frontline workers in maintaining access to HIV prevention and treatment as averting a potential rise in HIV transmission as expressed in the quotation below by a participant working in the eastern region of Ukraine:

Well, not anymore, because now harm reduction is working steadily, we are constantly organizing deliveries, and we quickly organized ourselves then. Well, I won't say that this is a large number of people who are infected, because, again, we quickly organized, there were social workers with their own cars who delivered distribution materials, delivered PrEP, delivered ART, and, so, the situation leveled off…. (East)

### 3.3 Continuing to work despite a lack of adequate financial compensation

Throughout programme adaptions during the ongoing war, organizations continued to provide services despite working for outdated pay scales that did not adequately reflect their work, even prior to the onset of the war. As one participant stated: “Well, I don't know how to tell a person that he should sit for 8 hours a day, the whole week, for 6500 UAH [approx. 224 USD]”. Participants spoke of the stress and burden that inadequate compensation added to their lives while they tried to maintain services during ongoing bombings and blackouts and without transportation and clean drinking water. Participants discussed the challenges to fulfil their work roles, burdensome reporting expectations from donors that continued throughout war, inadequate pay for frontline workers and the demoralizing effect this had on their work.

….To work for a social worker who can't earn her own bread. And other projects we can't give them. Well, we can give them something extra … Well, we, for example, take a social worker, there is a minimum salary. We can't go any higher than that. You make the rate 10,000 [UAH, approx. 345 USD]…. (South)

Participants spoke of delayed reviews of salary scales and outdated activity‐based compensation that no longer reflects current realities. At a time when frontline workers are taking on additional work to support their clients, there is a demand to fairly compensate them for their service and a fear that it will be harder to engage frontline workers to continue to provide these types of services without adequate pay.

…. a person took everything he or she needed, left the house, spent money on travel, came to his or her job. And this is the person who is ready to provide a service. The person who is looking for clients, who spends time. And if, well, I'm more in favour of such a system, maybe a bonus system. Because I understand that this system scares a lot of people and makes them quit. I know people who, when they went from being great, cool social workers to quality social workers, who are focused on their work, they just left. Because they didn't see their value in it. And of course, if we take the system in general, and what is paid for in prevention programmes, we understand that a social worker does more. That is, we do a syphilis test there, but we never get paid for it. When we ask why, they say, “Well, you just take it as a workload, [it isn't] hard for you?” Well, a person does this work, he spends time on it. (National)

While participants continue to work despite these delays in payments, this also poses questions for prevention programme funding post war, when international donors again pull out and the government re‐assumes responsibility for funding the HIV programme. Under the current condition of war, participants expressed concerns for the sustainability of staffing these services. As one participant stated:

…Yes, there are some projects left, or its prospects. And the prospects of it all are already kind of well…clear. That another year of these situations, a year of this war and the conditions of the real economic state of the country, m‐m‐m. … Unfortunately, I do not think that we will have very many people willing to provide such services. (South)

## 4 DISCUSSION

NGOs play a crucial role in service delivery for key population groups in Ukraine, particularly in the context of the ongoing war and displacement. These organizations serve as “first responders” in the HIV response [34], leveraging their proximity to communities, funders, and flexible models of practice to provide essential services and adapt to the changing conditions brought on by war. NGOs are often deeply embedded within the communities they serve, establishing trust and rapport with their clients. This closeness allows them to understand the specific needs and challenges faced by communities, tailoring their efforts accordingly. Particularly in the field of HIV prevention and treatment, these relationships prove invaluable, as they enable NGOs to reach individuals who may not otherwise have access to vital information and resources. Moreover, NGOs are characterized by their agility and innovation in service provision. They have the capacity to swiftly respond to emerging needs and adapt their programmes to address evolving circumstances, such as those created by war. In the context of Russia's war in Ukraine, NGOs have demonstrated remarkable resilience, adjusting their strategies to continue providing HIV prevention services amidst unimaginable disruptions.

However, it is impossible to ignore the impact that migration and ongoing conflict have on NGOs, affecting operational hours, the availability and location of services and staffing. Some organizations, previously operating region‐wide, have restricted their services due to logistical challenges in areas affected by conflict. Medical supply disruptions and limitations in available healthcare services have further exacerbated these challenges, which are widespread across various sectors in Ukraine, influenced by the geographical proximity to war zones and the intensity of hostilities in the region. Despite these challenges, our study demonstrates that NGOs, through their “dense connections” [34, p.5] to people, other organizations, government and funders, find themselves positioned as “first responders”, adapting their services as best as possible. Our findings align with Friedman et al.’s work highlighting how NGOs providing HIV services immediately adapted their responses following the escalation to full‐scale war in Ukraine, despite frontline workers also being impacted and, at times, displaced themselves [37]. Participants in our study recounted how their organizations worked to continue to make HIV prevention commodities and medicines available to key populations while expanding services to provide humanitarian aid to both clients and the wider community. Incredibly, some reports indicate increased enrolment in PrEP and some ART programmes in Ukraine since the start of the war [39, 40]. However, access to ongoing HIV treatment in occupied territories remains precarious, with a recent report indicating that ART access in the Donbas is now predicated on adopting a Russian passport—a condition that nearly half of PLHIV in the region are loathe to accept [41].

Despite the important role played by frontline workers, they remain underpaid, often volunteering their time while placing their own lives and mental health at risk. Our findings point towards how overworked and under‐resourced organizations across Ukraine continue to mobilize supports under extraordinary conditions. We wholeheartedly echo Roels et al.’s assertion that sustainable funding for organizations “are actually investments in disaster preparedness” and that “city/state governments should recognize the work of community organizations, learning that social support and collective care cannot occur without the pre‐existing structures that are well‐embedded and trusted within communities” [34, p.14].

We further stress the importance of including NGO representatives in important policy conversations at local, regional and national levels, as they contribute invaluable contextual and experiential knowledge and are best situated to understand the needs of their communities. During the war, we have seen how NGOs expanded their service delivery beyond their clients to respond to the needs of entire impacted communities. While we do not often turn to key population NGOs as first responders in humanitarian crises, their inherent adaptiveness is ideally suited to assume this role, as they hold important insights on community needs and movements, especially among the most marginalized populations [34, 40].

Critical questions remain around the sustainability of this current situation. How long will frontline workers be able to provide supports without adequate compensation for their work, which has only grown since the war? While further financial supports are urgently needed for frontline workers, ongoing, stable funding will be critical following the war. While currently recognizing the uncertainty of the postwar period, we maintain that there will be an urgent need for social and health services provided by NGOs and frontline workers [37]. We argue that the voices of frontline workers should be heard at policy‐ and programme‐planning tables to help rebuild communities affected by war, as well as to ensure that HIV care, harm reduction, HRT and other essential services for key populations are maintained.

### 4.1 Limitations and strengths

Our findings are based on key informant interviews and may not be generalizable to the experiences of all those working in NGOs in Ukraine. As these were key informant interviews, we did not collect detailed demographic data. However, our representation from those working in NGOs across the country, including in territories facing occupation, ensured that we are presenting a wide range of experiences. As our interviews occurred a year into the war, responses might be subject to recall bias. However, the incredibly sensitive nature of the discussions and our previous research relationships allowed for an opportunity to explore how NGOs responded to and adapted their services.

## 5 CONCLUSIONS

NGOs in Ukraine play a vital role as responders during structural shocks, such as war, adapting their services to meet the emergent needs of affected communities. There is an urgent need to support Ukrainian NGOs with adequate resources for key population service delivery—including HIV prevention and treatment—to facilitate a responsive and community‐centred approach. NGOs’ ability to operate closely with affected populations, coupled with their adaptability in the face of adversity, underscores their indispensability in safeguarding health, particularly in times of crisis.

## COMPETING INTERESTS

The authors declare that they have no competing interests.

## AUTHORS’ CONTRIBUTIONS

All authors supported the design of the study. DP, AG and TT collected the data. LL and LMM conducted the analysis. LL, LLM and NH wrote the first draft of the manuscript. All authors reviewed and approved the final version of the manuscript.

## FUNDING

This study is funded by the Canadian Institutes of Health Research [Funding Reference Number PJT‐148876]. The funding source had no design in the study nor in the collection, analysis or interpretation of the data. Robert Lorway is supported by a Tier 2 Canada Research Chair in Global Intervention Politics and Social Transformation.

## Data Availability

As the datasets involve sensitive data from criminalized and stigmatized groups, data will not be made publicly available. Some data may be made available from the corresponding author on reasonable request and approval from the NGOs and study team.

## References

[1] Roborgh S , Coutts AP , Chellew P , Novykov V , Sullivan R . Conflict in Ukraine undermines an already challenged health system. Lancet North Am Ed. 2022; 399(10333):1365–1367. 10.1016/S0140-6736(22)00485-8 35286845

[2] Zaliska O , Oleshchuk O , Forman R , Mossialos E. Health impacts of the Russian invasion in Ukraine: need for global health action. Lancet North Am Ed. 2022; 399(10334):1450–1452. 10.1016/S0140-6736(22)00615-8 35367006

[3] WHO . Surveillance system for attacks on healthcare (SSA). 2024. Available from: https://extranet.who.int/ssa/Index.aspx

[4] Tokar A , Osborne J , Slobodianiuk K , Essink D , Lazarus JV , Broerse JE . ‘Virus Carriers’ and HIV testing: navigating Ukraine's HIV policies and programming for female sex workers. Health Res Policy Syst. 2019; 17(1):23. 30819203 10.1186/s12961-019-0415-4 PMC6394058

[5] Kyselyova G , Martsynovska V , Volokha A , Nizova N , Malyuta R , Judd A , et al. Young people in HIV care in Ukraine: a national survey on characteristics and service provision. F1000Res. 2019; 8:323. 31105935 10.12688/f1000research.18573.1 PMC6498744

[6] Alliance for Public Health . Statistics. 2024. Available from: https://aph.org.ua/en/resources/statistics/

[7] U.S. Department of State . Where we work—PEPFAR. 2024. Available from: https://www.state.gov/where‐we‐work‐pepfar/

[8] The Global Fund . Ukraine. 2024. Available from: https://data.theglobalfund.org/location/UKR/overview

[9] Derksen J , Pavlova D , McClarty LM , Balakireva O , Herpai N , Lazarus L , et al. Awareness and utilization of HIV testing and prevention services among female sex workers in Dnipro, Ukraine: implications for prevention program strengthening from the Dynamics Study. Front Reprod Health. 2022; 4:879191. 36303675 10.3389/frph.2022.879191 PMC9580627

[10] Ministry of Health Ukraine . National Health Reform Strategy for Ukraine 2015–2020. 2015. Available from: https://en.moz.gov.ua/uploads/0/16‐strategy_eng.pdf

[11] Ministry of Health Ukraine . Healthcare reform: key steps to transforming Ukrainian healthcare. 2024. Available from: https://en.moz.gov.ua/healthcare‐reform

[12] Cairns G. HIV services in Ukraine resilient but starting to feel the strain. NAM Publications (aidsmap). 2023. Available from: https://www.aidsmap.com/news/oct‐2023/hiv‐services‐ukraine‐resilient‐starting‐feel‐strain

[13] U.S. Department of State . PEPFAR investing US $13 million to reach Ukrainian in need with life‐saving HIV treatment. 2022. Available from: https://www.state.gov/pepfar‐investing‐us‐13‐million‐to‐reach‐ukrainians‐in‐need‐with‐life‐saving‐hiv‐treatment/#:~:text=Over%20the%20past%20two%20months,for%20up%20to%20a%20year

[14] U.S. Department of State . PEPFAR moves swiftly to ensure life‐saving HIV services reach Ukraine. 2022. Available from: https://www.state.gov/pepfar‐moves‐swiftly‐to‐ensure‐life‐saving‐hiv‐services‐reach‐ukraine

[15] The Global Fund . Global fund announces additional emergency funding in Ukraine after one year of war. 2023. Available from: https://www.theglobalfund.org/en/news/2023/2023‐02‐21‐global‐fund‐announces‐additional‐emergency‐funding‐in‐ukraine‐after‐one‐year‐of‐war

[16] Holt E . Ukraine invasion: 6 months on. Lancet North Am Ed. 2022; 400(10353):649–650. 10.1016/S0140-6736(22)01635-X 36030807

[17] UNAIDS . SITREP: UNAIDS’ response to the crisis in Ukraine. 2023. Available from: https://www.unaids.org/sites/default/files/media_asset/Ukraine‐SitRep.pdf

[18] WHO . Emergency in Ukraine: external situation report #19. 2022. Available from: https://www.who.int/publications/i/item/WHO‐EURO‐2022‐5152‐44915‐65715

[19] WHO . War in Ukraine: situation report from WHO Country Office in Ukraine. 2023. Available from: https://iris.who.int/bitstream/handle/10665/371967/WHO‐EURO‐2023‐5319‐45083‐70235‐eng.pdf?sequence=1

[20] Holt E . Difficult choices for people with HIV in the Donbas. The Lancet HIV. 2024. 10.1016/S2352-3018(24)00006-7 38211597

[21] Public Health Center of the Ministry of Health of Ukraine. HIV‐infection in Ukraine: informational bulletin. 2022; 53:117.

[22] WHO . On the frontline of the fight against HIV: Ukraine's resilience and WHO's support. 2023. Available from: https://www.who.int/europe/news/item/19‐12‐2023‐on‐the‐frontline‐of‐the‐fight‐against‐hiv–ukraine‐s‐resilience‐and‐who‐s‐support

[23] The Global Fund . War in Ukraine: maintaining lifesaving HIV and TB services. 2024. Available from: https://www.theglobalfund.org/en/ukraine

[24] UNDP . AIDS and war: how Ukraine is combating HIV/AIDS in 2022. 2022. Available from: https://www.undp.org/ukraine/news/aids‐and‐war‐how‐ukraine‐combatting‐hiv/aids‐2022

[25] Vitek CR , Čakalo JI , Kruglov YV , Dumchev KV , Salyuk TO , Božičević I , et al. Slowing of the HIV epidemic in Ukraine: evidence from case reporting and key population surveys, 2005–2012. PLoS One. 2014; 9(9):e103657. 25251080 10.1371/journal.pone.0103657 PMC4174506

[26] BBC . Ukraine in maps: tracking the war in Russia. BBC; 2023. Available from: https://www.bbc.com/news/world‐europe‐60506682

[27] Public Health Center of the Ministry of Health of Ukraine. HIV‐infection in Ukraine: informational bulletin. 2023; 54:84.

[28] Duchenko A , Deshko T , Braga M. Crisis management by HIV/AIDS non‐governmental organisations in the post‐Euromaidan Ukraine led to opening new horizons. Drugs Alcohol Today. 2017; 17(3):149–156.

[29] Kravchenko N , Denisiuk O , Kuznetsova J , Jayaraj J , Zachariah R , Smyrnov P . Engaging people who inject drugs and their peers in HIV testing and harm reduction in Ukraine: do they make a difference? Journal Infect Dev Ctries. 2019; 13(7.1):118S–125S. 32065814 10.3855/jidc.11293

[30] Kuznetsova J , Sereda Y , Denisiuk O , Kravchenko N , Jayaraj J , Smyrnov P , et al. Linking intravenous drug users to treatment through non‐governmental organizations in Ukraine: how well is it working? Journal Infect Dev Ctries. 2019; 13(7.1):95S–102S. 32065812 10.3855/jidc.11291

[31] Trickey A , Semchuk N , Saliuk T , Sazonova Y , Varetska O , Walker JG , et al. Has resourcing of non‐governmental harm‐reduction organizations in Ukraine improved HIV prevention and treatment outcomes for people who inject drugs? Findings from multiple bio‐behavioural surveys. J Int AIDS Soc. 2020; 23(8):e25608. 32851812 10.1002/jia2.25608 PMC7450208

[32] Public Health Center of the Ministry of Health of Ukraine . World AIDS Day: “Leadership to communities!” [Internet, translated from Ukrainian]. Public Health Center. Ministry of Health of Ukraine. 2023 [cited 2024 Apr 26]. Available from: https://phc.org.ua/news/vsesvitniy‐den‐borotbi‐zi‐snidom‐liderstvo‐spilnotam

[33] Becker M , Balakireva O , Pavlova D , Isac S , Cheuk E , Roberts E , et al. Assessing the influence of conflict on the dynamics of sex work and the HIV and HCV epidemics in Ukraine: protocol for an observational, ethnographic, and mathematical modeling study. BMC Int Health Human Rights. 2019; 19:1–9. 10.1186/s12914-019-0201-y PMC6528269 31109323

[34] Roels NI , Estrella A , Maldonado‐Salcedo M , Rapp R , Hansen H , Hardon A . Confident futures: community‐based organizations as first responders and agents of change in the face of the Covid‐19 pandemic. Soc Sci Med. 2022; 294:114639. 34998135 10.1016/j.socscimed.2021.114639 PMC8683095

[35] Friedman SR , Rossi D , Braine N . Theorizing “Big Events” as a potential risk environment for drug use, drug‐related harm and HIV epidemic outbreaks. Int J Drug Policy. 2009; 20(3):283–291. 19101131 10.1016/j.drugpo.2008.10.006

[36] Friedman SR , Mateu‐Gelabert P , Nikolopoulos GK , Cerdá M , Rossi D , Jordan AE , et al. Big Events theory and measures may help explain emerging long‐term effects of current crises. Glob Public Health. 2021; 16(8‐9):1167–1186. 33843462 10.1080/17441692.2021.1903528 PMC8338763

[37] Friedman SR , Smyrnov P , Vasylyeva TI . Will the Russian war in Ukraine unleash larger epidemics of HIV, TB and associated conditions and diseases in Ukraine? Harm Reduct J. 2023; 20(1):119. 37658448 10.1186/s12954-023-00855-1 PMC10472698

[38] Lorway R , Thompson LH , Lazarus L , du Plessis E , Pasha A , Fathima Mary P , et al. Going beyond the clinic: confronting stigma and discrimination among men who have sex with men in Mysore through community‐based participatory research. Crit Public Health. 2014; 24(1):73–87.

[39] Cairns G . Rapid scale‐up of PrEP in Ukraine this year, despite the war. NAM Publications (aidsmap). 2022. Available from: from: https://www.aidsmap.com/news/oct-2022/rapid-scale-prep-ukraine-year-despite-war

[40] Lopatina Y , Żakowicz AM , Shabarova Z , Ford T , Fonseca FF , Odoke W , et al.. Safeguarding HIV prevention and care services amidst military conflict: experiences from Ukraine. BMJ Glob Health. 2023; 8(12):e014299. 10.1136/bmjgh-2023-014299 PMC10753732 38148111

[41] Holt E . Difficult choices for people with HIV in the Donbas. The Lancet HIV. 2024. 10.1016/S2352-3018(24)00006-7 38211597

